# The influence of plastic pyrolysis oil on fuel lubricity and diesel engine performance

**DOI:** 10.1039/d3ra08150h

**Published:** 2024-03-26

**Authors:** Anupap Pumpuang, Niti Klinkaew, Khatha Wathakit, Aunna Sukhom, Ekarong Sukjit

**Affiliations:** a Institute of Research and Development, Suranaree University of Technology Muang Nakhon Ratchasima 30000 Thailand; b School of Agricultural Engineering, Institute of Engineering, Suranaree University of Technology Muang 30000 Nakhon Ratchasima Thailand; c School of Mechanical Engineering, Institute of Engineering, Suranaree University of Technology Muang Nakhon Ratchasima 30000 Thailand ekarong@sut.ac.th

## Abstract

This study investigates the viability of using plastic oils derived from High-density polyethylene (HDPE), Polypropylene (PP), and Polystyrene (PS) as alternative fuels for diesel engines. The research focuses on comparing the physical and chemical properties, fuel lubricity, engine performance, combustion characteristics, and exhaust emissions of these plastic oils. Analysis revealed that PS exhibits different fuel properties compared to diesel, with a carbon range distribution similar to gasoline, while HDPE and PP properties closely resemble diesel fuel. To prevent potential engine damage, PS was excluded from engine tests. PP displayed the best fuel lubricity, attributed to its higher kinematic viscosity and sulphur content, reducing direct friction. Diesel followed, with PS and HDPE in decreasing order of lubricity. Diesel's lubricity was influenced by the 7% palmitic methyl ester content in the fuel. In engine tests, HDPE demonstrated BTE similar to diesel, while PP exhibited lower BTE due to combustion retardation, leading to increased energy losses and higher BSFC. The combustion characteristics, in-cylinder pressure, and heat release rate of HDPE closely resembled diesel, while PP showed significantly delayed combustion due to low oxygen content and higher kinematic viscosity. Notably, NO_*X*_ emissions from PP were lower than diesel and HDPE at all engine loads due to heat losses, resulting in a low in-cylinder temperature unsuitable for NO_*X*_ emission. HDPE produced higher NO_*X*_ emissions than diesel at low and middle loads due to its higher H/C ratio, promoting high thermal NO_*X*_ formation. HC emissions from both HDPE and PP were higher than diesel due to increased fuel supply, hindering chemical bond breakdown. Similarly, CO emissions increased for HDPE and PP due to insufficient time for complete combustion, with HDPE producing more CO due to its heavy composites and lower cetane index. Smoke emissions from both HDPE and PP surpassed diesel, attributed to lower oxygen and higher sulphur content, leading to increased sulphurate particulate matter formation, and lower fuel density referring to the high amount of fuel supplied to the engine.

## Abstract 2

A comprehensive study assesses the feasibility of utilizing plastic oils from HDPE, PP, and PS as diesel alternatives. The investigation concentrates on a comparison of fuel lubricity, engine performance, combustion and emission characteristics.

## Graphical abstract

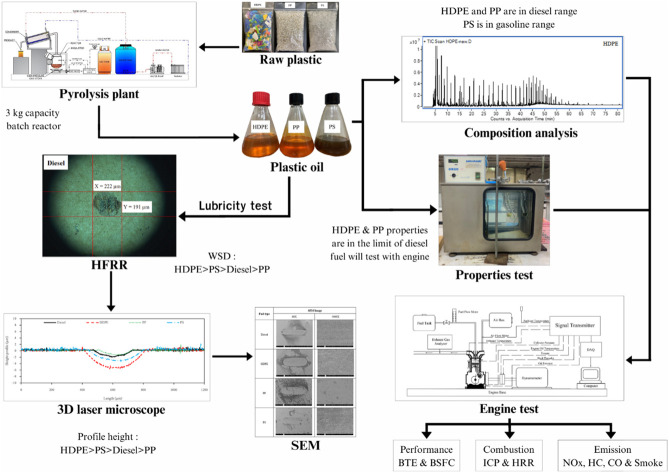

## 1 Introduction

Plastic, a versatile and widely used material, has become an integral part of modern society. It has revolutionized industries and transformed various aspects of human life. However, the widespread use and disposal of plastic have also led to significant environmental challenges. Plastics are derived from petrochemicals, which are primarily sourced from fossil fuels such as crude oil and natural gas.^1^ The production of plastic involves a complex process that includes polymerization, where small molecules called monomers are chemically bonded together to form long chains known as polymers. These polymers can be molded, shaped and fabricated into various products, making plastic highly adaptable for numerous applications.^2^ The production and consumption of plastics have skyrocketed over the past few decades, driven by factors such as population growth, urbanization and industrialization. The convenience, durability and affordability of plastic have contributed to its widespread use in packaging, construction, electronics, automotive parts and many other industries. However, the exponential growth in plastic production has led to significant environmental consequences. Plastic waste is a major concern, as it is non-biodegradable and can persist in the environment for hundreds of years. Improper disposal and inadequate recycling infrastructure have resulted in plastic pollution in landfills, oceans, rivers and ecosystems worldwide.^3,4^

To address this issue, the processing of waste plastics through pyrolysis, a thermal decomposition method, has gained attention. Pyrolysis is the process in which plastic is subjected to high temperatures, typically in the range of 300 °C to 500 °C, in a specialized reactor. The absence of oxygen prevents the plastic from burning and instead triggers a chemical breakdown, resulting in the production of various valuable byproducts. The waste plastic pyrolysis process leads to the conversion of plastic waste into three main products: liquid fuel, gas and solid residue. The liquid fuel is commonly referred to as plastic pyrolysis oil or plastic oil (PO).^4–7^ It possesses similar properties to conventional fuels and can be used as an alternative fuel source in various applications, including diesel engines, industrial boilers, or heating systems. The gas generated during the process can be used as a source of heat and energy. The solid residue, often called char or carbon black, can be further processed or utilized as a raw material in other industries such as vehicle tire production.^8,9^ Plastic oil has the potential to be used as a substitute for conventional diesel fuel. The quantity of PO from conversion and its properties depends on two main factors: (1) the composition of plastic feedstocks used in the pyrolysis process and (2) the pyrolysis conditions such as temperature, reaction time, catalyst and heating rate. Different types of plastics, such as Polyethylene (PE), polypropylene (PP) and polystyrene (PS), have distinct chemical structures and characteristics. These variations in plastic feedstocks can influence the composition and quality of the resulting PO. The four particular elements, which are moisture content, fixed carbon, volatile matter and ash content, are the factors that influence PO yield.^10^ Volatile matter and ash content are the major factors that influence the liquid oil yield in the pyrolysis process. High volatile matter feedstock provides more liquid oil yield, while high ash content feedstock provides less liquid yield while on the other hand it improves the ash yield.^11^ The pyrolysis yields from many research projects are shown in Table 1.

**Table 1:** Pyrolysis yield from different plastic feedstocks

Ref.	Plastic type	Reaction temperature (°C)	Catalyst type	Yield (%)		
				Liquid	Gas	Ash
5	HDPE	400	Non-catalyst	80.88	17.24	1.88
13	HDPE	550	Non-catalyst	84.7	16.3	—
12	HDPE	650	Silica	68.5	31.5	—
14	LDPE	500	Zeolite ZSM-5	95	5	—
13	LDPE	550	Non-catalyst	93.1	14.6	—
5	PP	400	Non-catalyst	69.82	28.84	1.34
16	PP	500	Non-catalyst	82.12	17.88	—
15	PP	740	Non-catalyst	48.8	49.6	1.6
17	PS	425	Non-catalyst	97	2.5	0.5
15	PS	600	Non-catalyst	89.5	10	0.5

Polyethylene is a commonly used plastic material that consists of a long chain of carbon atoms with two hydrogen atoms attached to each carbon atom. The two common kinds of PE are High-density polyethylene (HDPE) and Low-density polyethylene (LDPE). HDPE is a long, linear polymer chain with a high degree of crystallinity and low branching, which leads to high strength properties. According to Table 1, the liquid oil yield from HDPE was 80.88% at 350 °C, with reported efficiencies of 84.7% and 68.5% at temperatures of 550 °C and 650 °C, respectively.^12,13^ Similar to HDPE, LDPE is a long, linear polymer chain with a high degree of crystallinity, but has more low branching, which leads to lower tensile strength and hardness. From Table 1, a liquid oil yield of 95.0% was obtained at 500 °C,^14^ while another study reported a yield of 93.1% under similar conditions.^13^ Additionally, it is worth noting that PP, a saturated polymer with a linear hydrocarbon chain, exhibits excellent chemical and heat resistance, although it remains unmelted at temperatures below 160 °C. Liquid oil yields of 69.82% at 400 °C, 82.12% at 500 °C, and 48.8% at 740 °C were reported in studies conducted at different temperature conditions.^5,15,16^ PS is the polymer that has a structure consisting of a long hydrocarbon chain with a phenyl group attached to every other carbon atom. The liquid oil yields of 97% and 87.5% were obtained at 425 °C and 600 °C, respectively, as reported in previous studies.^15,17^ The increase in reaction temperature tends to decrease liquid oil yield, while the yield of gases was increased. This is due to liquid oil being cracked into gas form at higher temperature. Table 2 represents the element composition of each plastic pyrolysis oil. The PS pyrolysis oil has more carbon content than the others. High carbon content in pyrolysis oil refers to more oxygen being needed to provide a complete combustion reaction.

**Table 2:** Elemental composition of plastic pyrolysis oil a

Plastic type	C	H	N	O	S
HDPE	84.63	15.23	0.14	nd	nd
LDPE	84.93	14.18	0.08	nd	0.81
PP	84.62	15.23	0.14	nd	0.01
PS	91.63	8.31	0.06	nd	nd

a nd = not detected, ref. 25.

It is known that plastic pyrolysis oil has similar fuel properties to diesel fuel. The plastic pyrolysis oil properties are shown in Table 3. This results in neat plastic pyrolysis oil being useable as compression ignition engine fuel. In a recent study, it was observed that thermal efficiency decreased and fuel consumption increased when HDPE pyrolysis oil was employed, attributed to its lower gross calorific value compared to diesel fuel. Additionally, the lower viscosity of HDPE pyrolysis oil may result in a lower bulk modulus, potentially leading to increased fuel compressibility.^18^ For this reason, a high amount of fuel was injected into the combustion chamber that caused a higher rate of heat release and ignition retard. NO_*X*_ emission from using HDPE pyrolysis oil was increased compared with diesel fuel, due to the high amount of fuel that was injected, and HC and CO emission were increased due to the insufficient oxygen level during the combustion. The few oxygen molecules react with the carbon molecules within the low temperature combustion process. Table 4 shows engine performance, characteristics and emission from different plastic oil types and different research. For HDPE, a consistent pattern of engine performance was observed by several studies,^4,19,20^ demonstrating decreased brake thermal efficiency (BTE) and an increase in brake specific fuel consumption (BSFC). These trends were attributed to the fuel's low heating value, resulting in reduced energy output, and high kinematic viscosity, leading to inferior fuel atomization. In a study conducted in 2016, it was observed that the in-cylinder pressure (ICP) and heat release rate (HRR) were higher when compared to diesel fuel. This was attributed to delayed ignition caused by the low cetane index of PO, potentially resulting in poorer fuel–air mixing.^19^ Moreover, the high viscosity indicates low bulk modulus, which can cause combustion chamber fuel accumulation from more advanced injection timing. On the other hand, lower ICP and HRR were observed due to the reduction of ignition delay when comparing plastic oil with diesel fuel. Additionally, plastic oil contained moderate proportions of heavy carbon (C25 to C28) constituents, potentially necessitating a high heat of combustion and contributing to a low heat release rate.^20^ In terms of engine emissions, the decrease in smoke opacity of plastic oil could potentially be attributed to factors such as the absence of aromatic content, the presence of oxygenated moieties (alcohols), and the formation of premixed combustion mixture prior to ignition.^20^ The increase in NO_*X*_ due to the high peak pressure and temperature increases the formation of NO_*X*_. Also, the higher calorific value and high delay period increases the peak temperature, resulting in higher NO_*X*_. Increase in CO and HC due to the retarded ignition cause the fuel to have less time to mix with air and combust. Conversely, smoke emission was found to increase due to the high aromatic content in plastic oil (PO), low volatility, and high viscosity, leading to poor spray characterization and mixture formation, ultimately resulting in low combustion efficiency.^4,19^ From the result of low HRR, the NO_*X*_ emission was decreased due to lower in-cylinder temperature that is unsuitable for NO_*X*_ formation. CO and HC were decreased due to advanced ignition from the high cetane index of PO, causing advanced ignition which provided more time to combust. For the other type of PE, LDPE, found the same trend of engine performance and combustion characteristics. BTE was increased and BSFC was decreased due to the low heating value and high viscosity, which cause fuel to have worse atomization and less energy release. The decrease in ICP due to advanced injection from low bulk modulus causes advanced ignition, occurring further before top dead center than diesel fuel. In the emission section, relevant findings have been highlighted in various studies.^21,22^ It was observed that NO_*X*_ emissions decreased due to lower ICP and lower heating value, resulting in a low in-cylinder temperature unsuitable for NO_*X*_ emissions.^23^ CO and HC emissions were increased due to lower in-cylinder temperature and low cetane index providing a lack of heat to break the chemical bonds of the fuel and form CO_2_. For PP, it was observed that lower BTE and higher BSFC were found compared to diesel fuel, attributed to the low heating value and high viscosity, resulting in poorer fuel atomization and lower energy release during the combustion process.^24^ Low ICP and HRR due to significantly lower cetane index cause ignition retardation providing less time for air–fuel mixing. For emissions, NO_*X*_ was decreased due to low in-cylinder temperature. HC emission was increased due to higher aromatic compounds in LDPE oil requiring more energy to break hydrocarbon chains. CO was decreased due to LDPE not having low aromatic hydrocarbon content, but a short ignition delay period and high oxygen content that result in decreased CO emissions in comparison to diesel operation.

**Table 3:** Fuel properties of plastic pyrolysis oil

Properties	Unit	Feedstock				
		HDPE^a^	LDPE^b^	PP^a^	PS^a^	Diesel^a^
Higher heating value	MJ kg^−1^	41.2	42.4	42.9	38.1	42–46
Kinematic viscosity	mm^2^ s^−1^	6.54	3	1.25	1.16	2–4.5
Density@15 °C	g cm^−3^	0.82	0.844	0.79	0.90	0.82–0.85
Flash point	C	<20	—	<20	<20	61.5 min
Sulphur content	mg kg^−1^	—	—	7.82	15.56	10 max
Cetane index	—	—	47.99	—	—	—

a Ref. 26.

b Ref. 24.

**Table 4:** Engine performance compared with diesel fuel

References	Engine type	Engine performance			Combustion characteristic		Emission				Plastic type
		BTE	BSFC		ICP	HRR	Smoke	NO_*X*_	CO	HC	
4	4 stroke, 1-cylinder, DI, water cooled, naturally aspirated, 3.7 kW @ 1500 rpm	↓		↑	—	—	↑	↑	↑	↑	HDPE
19	4 stroke, 1-cylinder, DI, water cooled, naturally aspirated, 3.7 kW @ 1500 rpm	↓		—	↑	↑	↑	↑	↑	↑	HDPE
20	4 stroke, 1-cylinder, DI, water cooled, naturally aspirated, 4.4 kW	↓		↑	↓	↓	↓	↓	↓	↓	HDPE
22	4 stroke, 1-cylinder, DI, water cooled, naturally aspirated, 3.7 kW @ 3000 rpm	↓		↑	↓	—	—	↓	↑	↑	LDPE
21	4 stroke, 4-cylinder, DI, water cooled, naturally aspirated, 68 kW @ 1500 rpm	↓		↓	↓	—	—	↓	↑	↑	LDPE
23	4 stroke, 1-cylinder, DI, water cooled, naturally aspirated, 3.7 kW @ 3000 rpm	↓		↑	↓	—	↑	↓	↑	↑	LDPE
24	4 stroke, 4-cylinder, DI, water cooled, turbo charger, 68 kW @ 1500 rpm	↓		—	↓	↓	—	↓	↑	↑	PP

Despite their enduring popularity in the transportation and industrial sectors due to their substantial load carrying capacity and versatility, diesel engines necessitate the use of fuel for operation. The predominant source of this fuel stems from the petroleum industry, which raises sustainability concerns. The prospect of alternative fuel sources has become increasingly appealing. Notably, plastic oil fuel emerges as a compelling option, given its comparable internal composition to traditional diesel fuel. This similarity arises from the fact that both plastics and diesel fuel are derived from the petroleum industry. In this study the use of three types of plastic, which are the main plastic waste in Thailand, and were high-density polyethylene (HDPE), polypropylene (PP) and polystyrene (PS) to make pyrolysis oil as a compression ignition engine fuel were studied. Their three-chemical compositions, chemical and physical fuel properties, fuel lubricity, engine performance, combustion characteristic and emission characteristics were investigated.

## 2 Methodology

### 2.1 Production of plastic oil

**Fig. 1:**
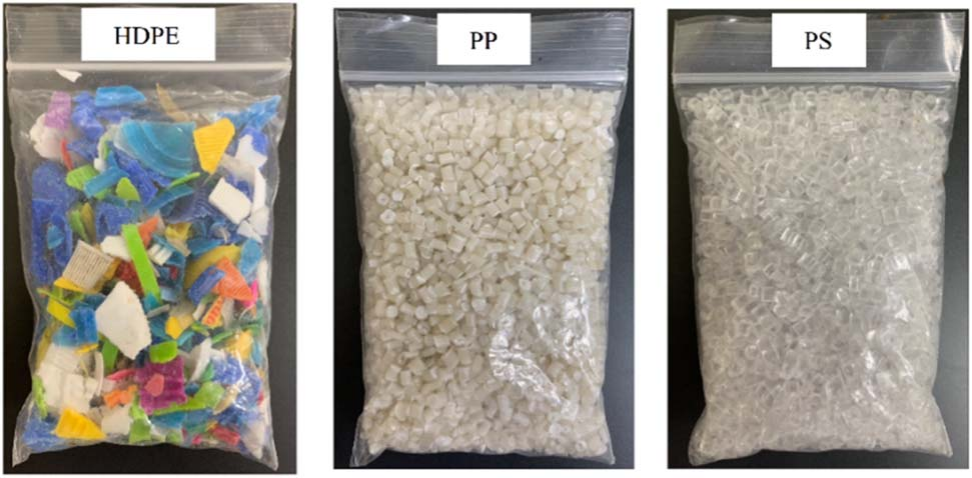
Raw plastic materials.

**Fig. 2:**
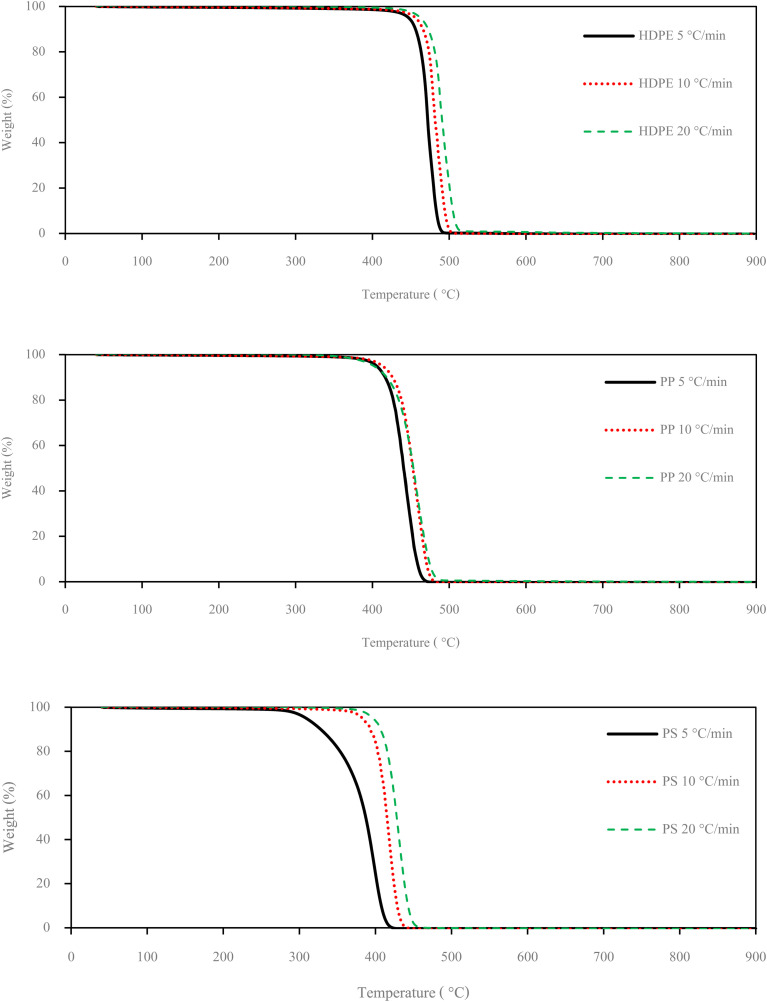
Decomposition curves of plastic raw materials at different heating rates.

**Fig. 3:**
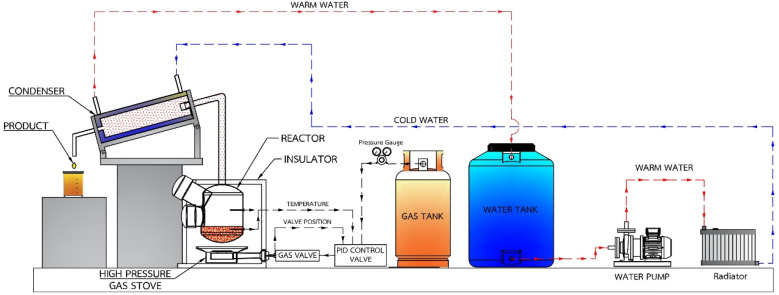
Schematic diagram of the plastic pyrolysis oil production.

**Fig. 4:**
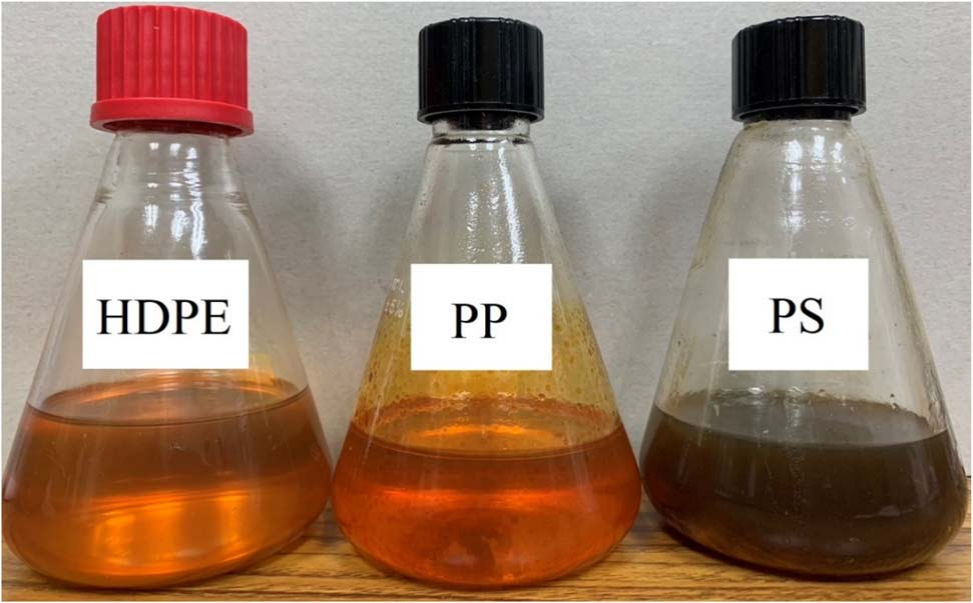
Plastic pyrolysis oil after from the pyrolysis process.

Small pieces (0.5–1.0 cm) of HDPE, PP and PS waste plastic were bought from Somkid Plastic Import and Export Company Limited, Fig. 1 shows the raw plastic materials used to produced pyrolysis oil. To examine the optimized pyrolysis temperature, the Mettler Toledo model TGA/dsc1 was used for thermogravimetric analysis (TGA). The conditions of TGA include a temperature range of 35 °C to 850 °C, 3 different heating rates (5 °C min^−1^, 10 °C min^−1^ and 20 °C min^−1^) and nitrogen flow rate of 5 L min^−1^. Fig. 2 shows the decomposition curves of HDPE, PP and PS with three different heat supplied rates. It can be seen from Fig. 2 that decreasing the heating rate caused early weight loss and reduced degradation temperature for all kinds of plastic. On the other hand, increasing the heating rate resulted in increased degradation temperature and late weight loss occurred. At a heating rate of 5 °C min^−1^, PS began to degrade at 280 °C and the end of degradation was at 440 °C. The starts of degradation temperature of HDPE and PP were 440 and 390 °C, respectively, and the ends of degradation temperature were 490 and 470 °C, respectively. For this reason, a 43.77 L chamber for lab scale pyrolysis bath reactor was used to produce the plastic pyrolysis oils. The raw waste plastic (3 kilograms) was put into the reaction chamber, then heated up to 200 °C with a heating rate of 10 °C min^−1^ and the temperature maintained for 40 minutes for the pre-pyrolysis phase. For the main-pyrolysis phase, the chamber was slowly heated up to 200–600 °C with a heating rate of 4 °C min^−1^ and the temperature was maintained for 6 hours to decompose solid plastic. Liquefied petroleum gas was used as the energy source of the heater for both pre-pyrolysis and main-pyrolysis phases with flow rate controlled by a PID gas controller. The gas from the plastic waste is sent through a condenser column to condense into liquid oil. The liquid oil was filtered and stored in a tank. Hot water from the water storage tank was pumped into a radiator to exchange heat and then flowed to the pyrolysis chamber with inlet temperature of 30 °C to cool down the chamber temperature and expel water at a temperature of 50 °C. The schematic diagram of the plastic pyrolysis oil production is shown in Fig. 3. The plastic pyrolysis oils derived from HDPE, PP and PS are shown in Fig. 4.

### 2.2 Gas chromatography and ultimate analysis

To identify the components of plastic oils, GC-MS was employed. The column for GC-MS analysis was a DB-wax capillary column (60 m length × 0.25 mm inner diameter, 0.25 μm film thickness). Helium was used as a carrier gas with a constant flow rate of 1.0 mL min^−1^. The oven temperature was programmed to operate from 70 °C to 250 °C, with an initial temperature of 70 °C that was held for 3 min, followed by a rate of heating of 3 °C min^−1^ to a temperature of 180 °C and then a rate of 10 °C min^−1^ to a final temperature of 250 °C, which was held for 25 min. The inlet was held at 250 °C with a split ratio of 20 : 1. The injection volume was 1 μL per sample. The mass spectrometer was scanned from mass to charge ratio (*m*/*z*) of 35 to 550 with the source at 250 °C. To identify the molecular compounds of plastic oil of PO, the ultimate analyzer model LECO 628 series was employed.

### 2.3 Fourier transform infrared (FTIR) spectroscopy

The chemical structural analysis of the sample was conducted employing a Bruker model Tensor 27 FTIR spectrometer, utilizing the attenuated total reflectance (ATR) technique. The analysis encompassed a spectral range spanning 450 to 4000 cm^−1^.

### 2.4 Fuel properties

The fuel properties are the parameters used to determine whether the fuel can be used in an engine.

In this study fuel properties tests were according to American Society for Testing and Materials (ASTM) standard. And diesel fuel used in this study is a blend of 7% biodiesel and 93% diesel according to the requirements of the Thailand Department of Energy.

### 2.5 Experiment setup of engine test

**Fig. 5:**
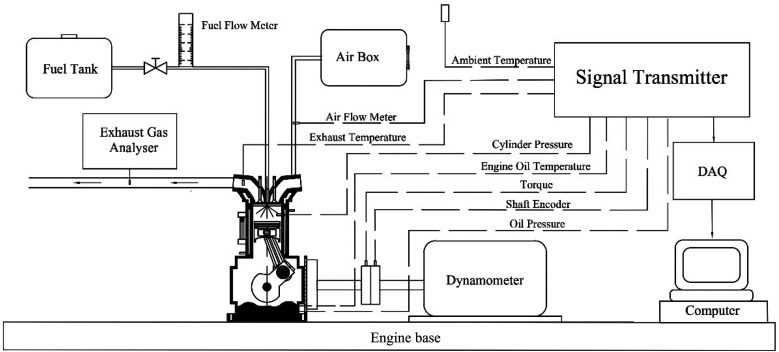
Schematic diagram of the experimental installation for the engine test.

Experiments were conducted using a single cylinder, four stroke diesel engine (KWM186FAE) with air cooling, natural aspiration, direct injection and rated power of 6.4 kW at 3600 rpm. The engine was operated at 2500 rpm as its usual operating speed with different loads: 25%, 50% and 75% of maximum torque. The engine loads were applied and measured using a hydraulic dynamometer. All the data were recorded after the engine had been operated constantly. The intake airflow rate, intake air temperature, and the exhaust gas temperature are attained from the standard engine test rig. In-depth analysis of the pressure trace and combustion characteristics necessitate the measurement of in-cylinder pressure. To achieve this, a Kistler 6052C pressure transducer was carefully installed for precise observation of the pressure trace. Prior to data acquisition, the pressure trace is amplified through a Kistler 5064C charge amplifier. Additionally, to establish the measured in-cylinder pressure with accuracy, the engine's crank position is essential and was determined using a Kistler crank angle encoder set (type 2614CK1). A schematic diagram of the experimental setup used in the study is shown in Fig. 5, and engine specifications are shown in Table 5. Gaseous emissions, including HC, CO and NO_*X*_, were quantified using a Testo 350 analyzer. Smoke emissions were assessed employing a Testo 308 analyzer. Throughout each experimental condition, the engine's stability was ensured through continuous monitoring of exhaust temperatures and exhaust emissions. The specifications of gaseous emission analyzers are shown in Table 6.

**Table 5:** Test engine specification

Engine data	4 stroke diesel engine
Engine model	KWM186FAE
Number of cylinders	1
Cooling system	Air cooled
Bore × stroke (mm × mm)	86 × 72
Displacement volume (cc.)	418
Rated power (kW rpm^−1^)	6.4/3600
Maximum torque (N m rpm^−1^)	18/2500
Compression ratio	19.5 : 1

**Table 6:** Gas analyzer specification

Parameter		Measuring technique	Measuring range	Resolution	Accuracy
**Testo 350**					
	NO	Chemiluminescence	0–4000 ppm	1 ppm	±5 < 100 ppm
	NO_2_	Chemiluminescence	0–500 ppm	0.1 ppm	±5 < 100 ppm
	CO	Nondispersive infrared	0–10,000 ppm	1 ppm	±10 < 200 ppm
	HC	Flame ionization detector	0–40,000 ppm	10 ppm	±400 ppm

**Testo 380**					
	Smoke index	Photodiode (filter paper)	0–6	0.1	±0.2

### 2.6 Fuel lubricity test

The High-Frequency Reciprocating Rig (HFRR) is a widely used testing apparatus for evaluating the lubricity of fuels, especially for diesel and biodiesel fuels. This test method assesses the ability of the fuel to minimize friction and wear between two metal surfaces, simulating the conditions within the fuel delivery systems of diesel engines. The HFRR used in this study was developed by PCS instruments and tests according to ISO 12156-1 (2018) standard test methods.^27^ Key components of the HFRR apparatus encompass the vessel containing the test fuel sample, the test friction assembly comprising a steel ball and disc, the electric sample heating unit, the load-bearing system, and the electromagnetic vibratory mechanism governing oscillatory motion at a defined frequency and stroke of the test ball holder. The mechanical system of the apparatus was enclosed within a cabinet, where environmental parameters such as the temperature and relative humidity of the surrounding air were measured at a distance of 15 cm from the friction interface. The HFRR system was outfitted with a control system linked to a computer featuring specialized HFRPC software. This software facilitated the input of essential measurement parameters and the archival of test results. Additionally, the HFRPC software provided the means for calculating the average friction coefficient and the average percentage of lubricating film thickness.

## 3 Results and discussion

### 3.1 Plastic fuel characterization

#### 3.1.1 Gas chromatography

**Fig. 6:**
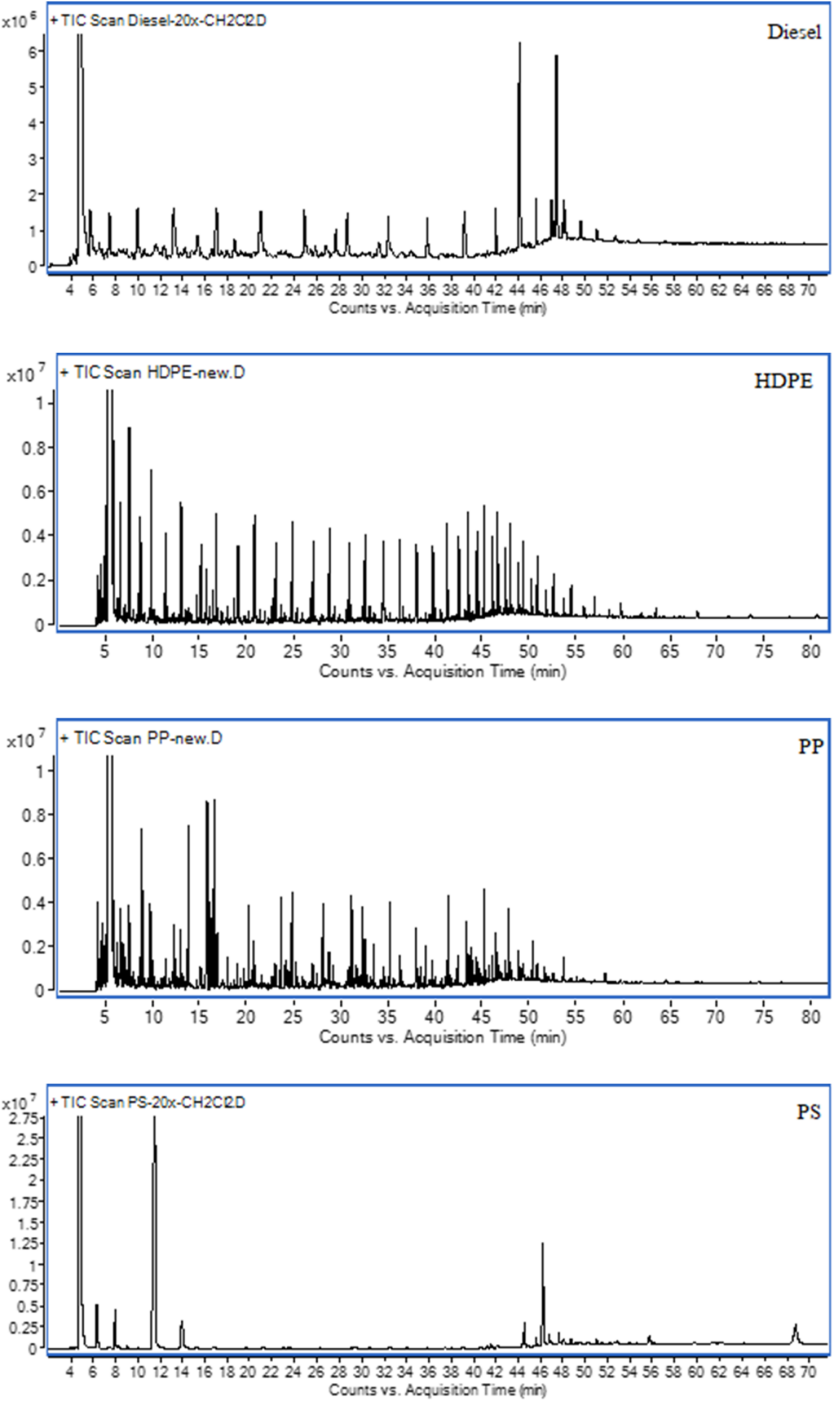
Total ion chromatograms from GC analysis of test fuels.

The GC peaks from GC-MS analysis of diesel and plastic oils are shown in Fig. 6 and the carbon range distribution of diesel and plastic oils are shown in Table 7. It can be seen from Table 7 that the main component of PS is in the range of C6 to C12 carbon range, which is the range of gasoline fuel, while HDPE and PP have the main components in the range of diesel fuel (C13 to C18). This due to their chemical structure, PS contains aromatic benzene rings in its structure, which can contribute to the formation of aromatic and gasoline-range hydrocarbons during pyrolysis. While PP and HDPE are aliphatic polymers, and they do not contain benzene ring or aromatic structures.

**Table 7:** Carbon range distributions of test fuels

Carbon number	Fuel type			
	Diesel	HDPE	PP	PS
C6–C12 (%)	12.091	26.959	31.142	72.543
C13–C18 (%)	51.519	41.322	50.848	19.918
C19–C23 (%)	30.594	21.702	14.665	7.539
>C23 (%)	5.796	10.017	3.346	—

#### 3.1.2 Ultimate analysis

Table 8 shows element composition of test fuel. It can be seen that diesel had contents of 83.230% of carbon, 11.020% of hydrogen, 1.427% of oxygen, 0.018% of nitrogen and no sulphur content. HDPE had contents of 85.004% of carbon, 12.549% of hydrogen, 1.38% of oxygen, and 0.2452% of sulphur. PP had contents of 85.535% of carbon, 13.541% of hydrogen, 0.6402% of oxygen and 0.1187% of sulphur. PS had contents of 87.913% of carbon, 6.42% of hydrogen. Diesel is a hydrocarbon-rich fuel with high carbon and hydrogen content and minimal sulfur, ensuring clean combustion. HDPE and PP, as polymers, show varying hydrocarbon compositions with oxygen and sulfur, suggesting potential combustion challenges and emissions. Polystyrene, abundant in carbon and lower in hydrogen, lacks sulfur data but may introduce unique combustion properties to a fuel mix. The limited sulfur in diesel and polymers is environmentally advantageous, reducing sulfur emissions during combustion.

**Table 8:** CHONS elemental composition of test fuels

Elements	CHONS elemental composition (wt%)			
	Diesel	HDPE	PP	PS
Carbon (C)	83.230	85.004	85.535	87.913
Hydrogen (H)	11.020	12.549	13.541	6.420
Oxygen (O)	1.427	1.380	0.6402	Not analyzed
Nitrogen (N)	0.018	Not detected	Not detected	Not detected
Sulphur (S)	0	0.2452	0.1187	Not detected

#### 3.1.3 FTIR result

**Fig. 7:**
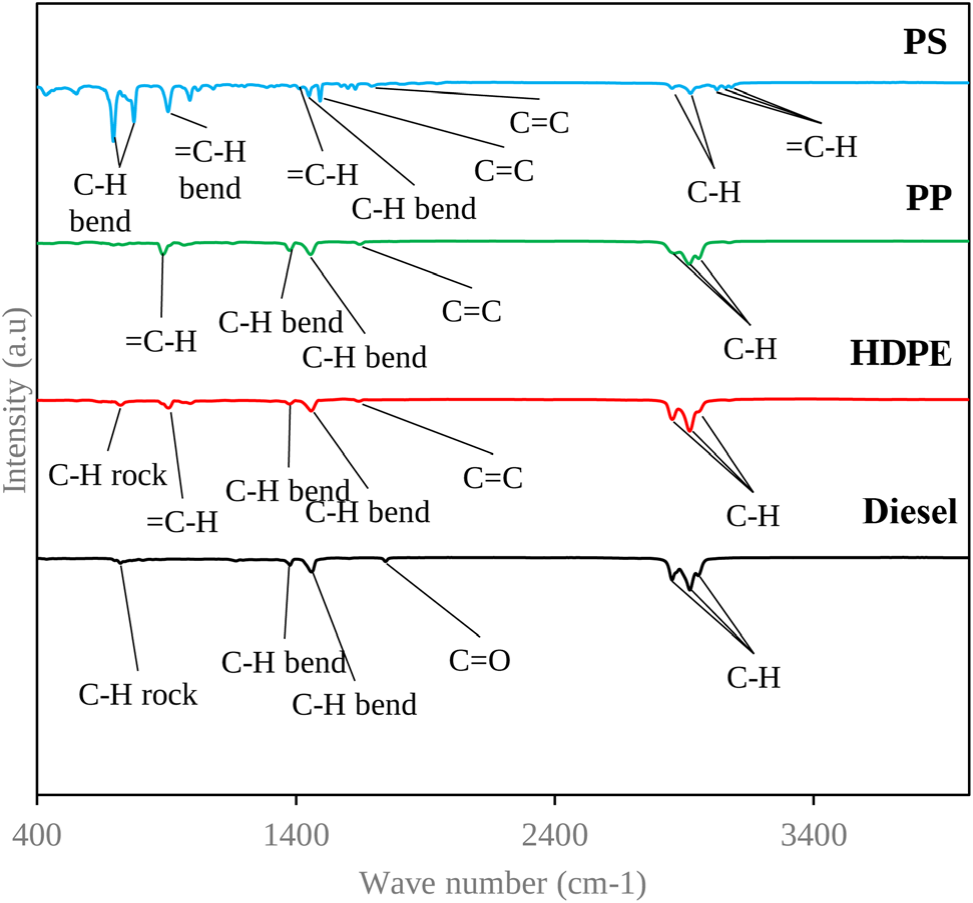
IR spectra of test fuels.

The IR spectra of four test fuels are shown in Fig. 7, which indicates that diesel, HDPE and PP provide a similar spectrum, while PS has significant differences. Table 9 shows the type of vibration and functional group in various positions of test fuels. The main functional group of diesel, HDPE and PP are alkane and alkene, while PS still has alkane and alkene groups, as well as an aromatic group.

**Table 9:** Identified functional groups present in test fuels

Wave number (cm^−1^)				Frequency of wavelength (cm^−1^)	Vibration type	Nature of functional group
Diesel	HDPE	PP	PS			
—	—	—	3082, 3059, 3026	3100–3000	=C–H stretch	Alkenes
2955	2957	2955	—	3000–2850	C–H stretch	Alkane
2922, 2854	2922, 2854	2918, 2858	2924, 2853	3000–2850	C–H stretch	Alkane
1745	—	—	—	1760–1740	Alkyl carbonate	Carbonyl
—	1639	1645	1630	1680–1620	Alkenyl C=C stretch	Alkene
—	—	—	1493	1600–1450	C=C stretch	Aromatic
1460	1458	1454	1450	1470–1450	C–H bend	Alkanes
—	—	—	1412	1415	=C–H bend	Alkenes
1377	1375	1377	1572	1590–1380	C–H bend	Alkanes
—	987, 908	966, 887	—	100–650	=C–H bend	Alkenes
721	721	—	—	730–675	C–H rock band	Alkenes
—	—	—	696	1000–650	=C–H bend	Alkenes
—	—	—	775	900–675	=C–H bend	Aromatic

#### 3.1.4 Fuel properties

**Fig. 8:**
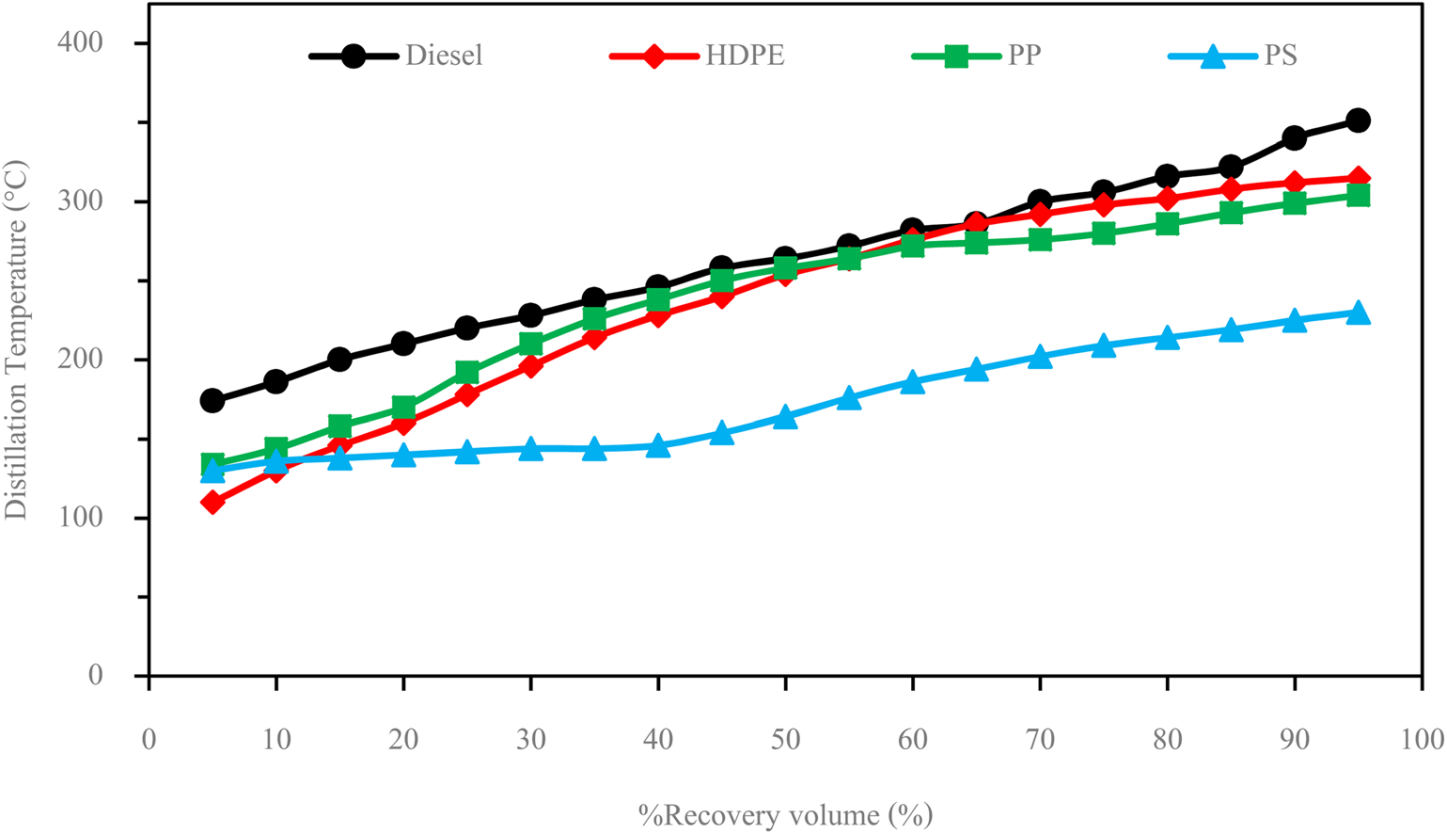
Distillation curves of test fuels.

The fuel properties, limits and test standards are shown in Table 10. It can be seen that fuel properties of HDPE and PP are in the limit of diesel fuel limit while all of PS properties are out of limit. Higher heating value of HDPE and PP are closer to diesel fuel while PS provides significantly lower higher heating value than diesel fuel due to its chemical composition that in the range of gasoline fuel. The kinematic viscosity of HDPE and PP are lower than diesel fuel, which can improve fuel atomization during combustion process, essential in the best fuel-to-heat conversion but it possible to provides worse fuel lubricity than diesel. While kinematic viscosity of PS is lower than diesel fuel limit. For specific gravity and density, the chemical composition which similar to diesel fuel introduced the similar in specific gravity and density of HDPE and PP. While PS content of high amount of shorter chain length hydrocarbon result in better packing efficiency that refer to higher in density and specific gravity. Due to the benzene ring and aromatic structure of PS introduced the higher volatile hydrocarbon of PS pyrolysis oil which can result in lower flash point of fuel. While the HDPE and PP are aliphatic polymers without aromatic structures which can provide lower volatile hydrocarbon than PS refer to higher in flash point of fuel. PP has a higher cetane index than diesel fuel, which can improve air–fuel mixing during the combustion process, while PS has a negative in the cetane index due to a significantly lower 50% recovery temperature. It can be seen from Fig. 8 that the distillation temperatures of HDPE and PP are close to diesel fuel due to their composition being in the range of diesel fuel, while PS has a significantly different distillation temperature than diesel fuel due to it having a composition in the range of gasoline fuel. With the significantly different in fuel properties of PS from diesel fuel, PS will not test in engine test section to prevent the damage the can be occur to the engine.

**Table 10:** Test fuel properties

Properties	Unit	Test standard	Limit	Fuel type			
				Diesel	HDPE	PP	PS
Higher heating value	MJ kg^−1^	D240	—	45.5424	45.7219	45.6843	41.2263
Kinematics viscosity	mm^2^ s^−1^	D445	1.8–4.1	3.05	2.51	2.69	1.63
Specific gravity at 15.6 °C	—	D1298	0.81–0.87	0.825	0.82	0.81	0.95
Density@15.6 °C	g cm^−3^	D1298	—	824.25	819.19	809.2	949.06
Flash point	°C	D445	>52	79	55	60	36
Cetane index	—	D976	>50	55.71	54.79	59.75	Not evaluated

### 3.2 Lubricity

**Fig. 9:**
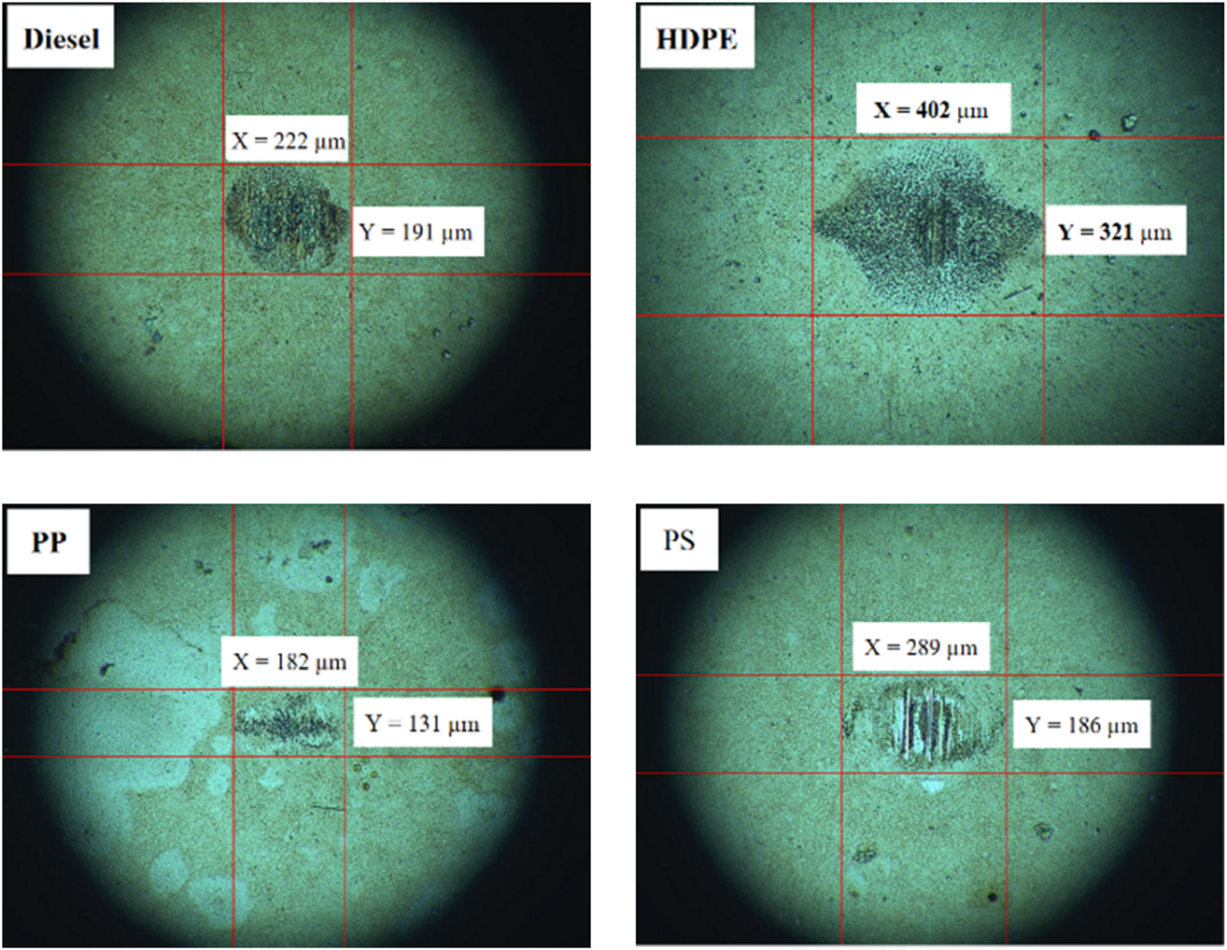
Wear scar diameters from HFRR.

**Fig. 10:**
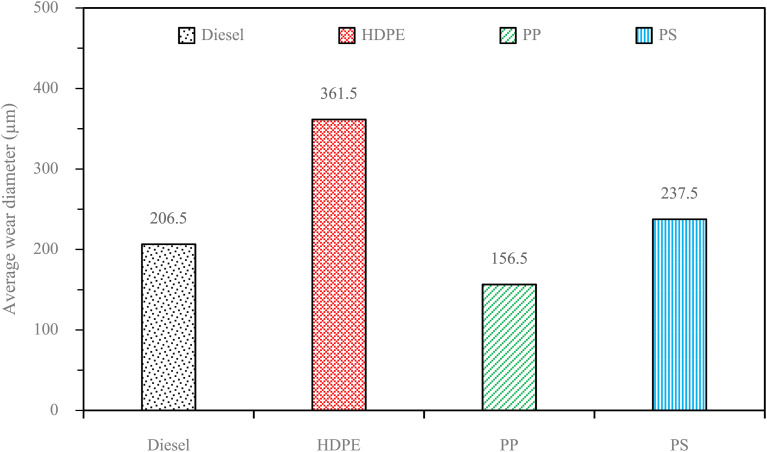
Average wear scar diameters of test fuels.

**Fig. 11:**
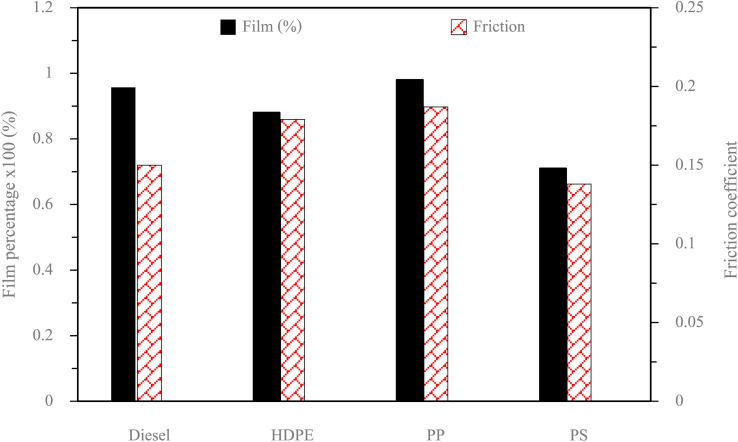
Average film percentage and friction coefficients of test fuels.

**Fig. 12:**
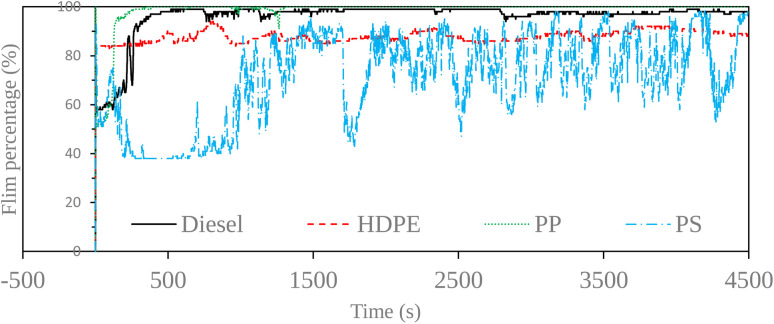
Film percentages at various times.

Fuel lubricity is a critical property in the context of engines and machinery, particularly those powered by diesel and other compression-ignition fuels. The wear scar diameters from HFRR tests of all test fuels are shown in Fig. 9, which represents the *X* axis and *Y* axis wear scar. Fig. 10 shows the average wear scar diameter, which indicates that PP provides the best lubrication followed by diesel, PS and HDPE in order. HDPE provides the worst fuel lubricity due to it having 10% less film percentage than PP, although both of them have the carbon distribution in the range of diesel fuel. That PP provides the best fuel lubricity may be due to the higher kinematic viscosity and sulphur content in PP providing a thicker film boundary, which can reduce direct contact between moving surfaces, steel ball and disc. For diesel fuel, the 7% biodiesel present in the diesel fuel can enhance fuel lubricity. Fig. 11 shows the average film percentage and average friction coefficient, which are parameters that have an inverse relationship with each other; a fuel having a higher film percentage will provide a low friction coefficient and low friction results in a small wear scar diameter. Considering Fig. 11, it is found that diesel, HDPE and PP are in the relationship described earlier while the PS is not. PS has a lower friction coefficient than diesel fuel, but provides a larger wear scar diameter than diesel fuel, which may be due to the main chemical component of PS being in the range of gasoline, which has lower evaporation temperature. The HFRR test is operated at 60 °C, which can evaporate the light components providing poor fuel lubrication, while PS in the test fuel results in heavy components that provide better lubrication, and only remain during the test. Fig. 12 shows the relationship between HFRR test time – film percentage and the relationship between HFRR test time–friction coefficient, respectively.

**Fig. 13:**
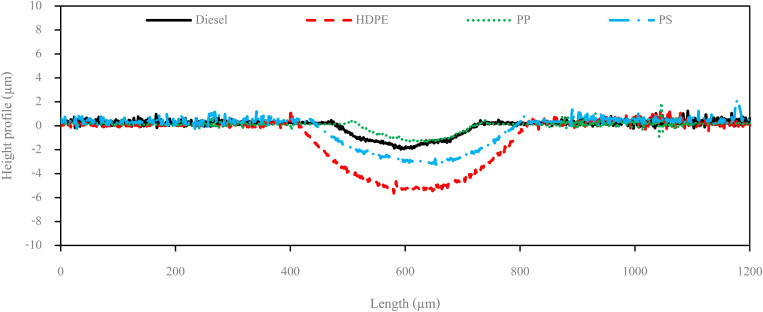
Wear scar depth on HFRR lower specimen.

**Fig. 14:**
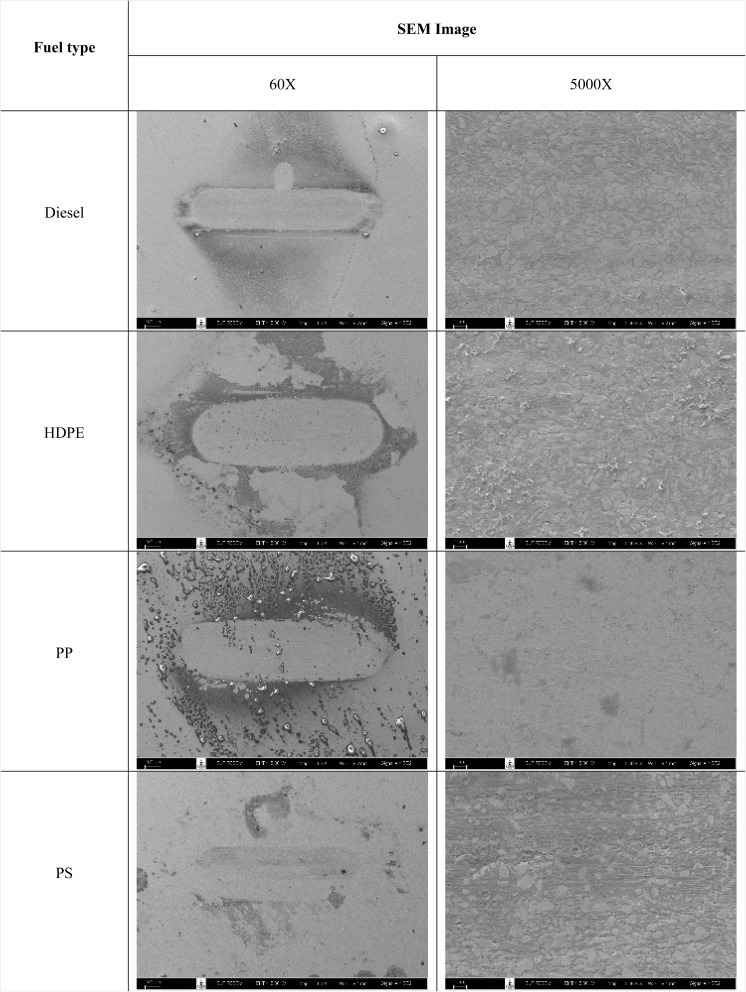
Images of worn area on the disc specimens taken by scanning electron microscopy.

Fig. 13 shows the wear scar depths of test fuels which were 2.865 μm, 7.256 μm, 3.946 μm and 3.680 μm under lubrication of the diesel, HDPE, PP and PS respectively and these correspond to the wear scar diameters. The main indicator for the diesel range fuels, diesel, HDPE and PP, is the kinematic viscosity, which is the inverse of wear scar depth. In the case of lower viscosity fuels, the boundary lubrication regime may prevail, where the thin fuel film between the surfaces may not provide sufficient separation to prevent direct contact. This condition can lead to increased friction and wear, resulting in deeper wear scars on the surfaces, while the lubricity of PS obtained from heavy components remains during the HFRR test and it should not be compared with the other three test fuels. Fig. 14 shows the worn area on the disc specimen from test fuels. PP provides a smoother worn area than other fuels but has a thick residue surface. Diesel and HDPE provide rough and quite thick residue surfaces, while PS has a rough worn area and is almost free of residue surface. Table 11 identifies the elements of the worn area of the disc specimen. The presence of carbon on the worn area may indicate the deposition of carbonaceous materials originating from the fuel. In general, increased carbon content can suggest the formation of protective carbon-based films or the accumulation of carbon residues, impacting the surface interactions and potentially influencing the wear characteristics of the HFRR disc. However, PP that has the smallest wear scar diameter, has the lowest carbon on the worn area. The presence of lower carbon content may indicate the formation of effective protective surface films or boundary layers that prevent direct metal-to-metal contact and minimize wear on the HFRR disc. These protective films can act as effective lubricants, reducing friction and wear, and thereby contributing to the smaller observed wear scar diameter, despite the lower carbon presence.

**Table 11:** Element content on the worn surface

Fuel type	Element weight percentage (%)			
	C	O	Cr	Fe
Diesel	8.71	2.11	3.01	86.17
HDPE	11.71	3.91	0	84.38
PP	5.22	2.05	1.72	90.74
PS	24	1.83	3.85	70.32

Moreover, the FTIR spectrum of the PP showed more alkenes than the other three fuels, which provide worse fuel lubricity. Alkenes contain at least one carbon–carbon double bond, which introduces a degree of reactivity into their molecular structure. This reactivity can make alkenes less effective as lubricants compared to alkanes. Additionally, alkenes may be more prone to oxidation and degradation, further reducing their lubricating properties.^28^ The other three test fuels have alkanes as the main functional group. Alkanes typically provide better fuel lubricity compared to alkenes. This is because alkanes have a saturated hydrocarbon structure with only single covalent bonds between carbon atoms, making them more stable and less reactive. This stability allows alkanes to form more robust boundary lubricating films on engine surfaces, reducing friction and wear.^28^ Furthermore, the appearance of the ester group (C=O) of diesel fuel, which forms 7% of the biodiesel in diesel fuel, can enhance the fuel lubricity.^29^

### 3.3 Engine performance

**Fig. 15:**
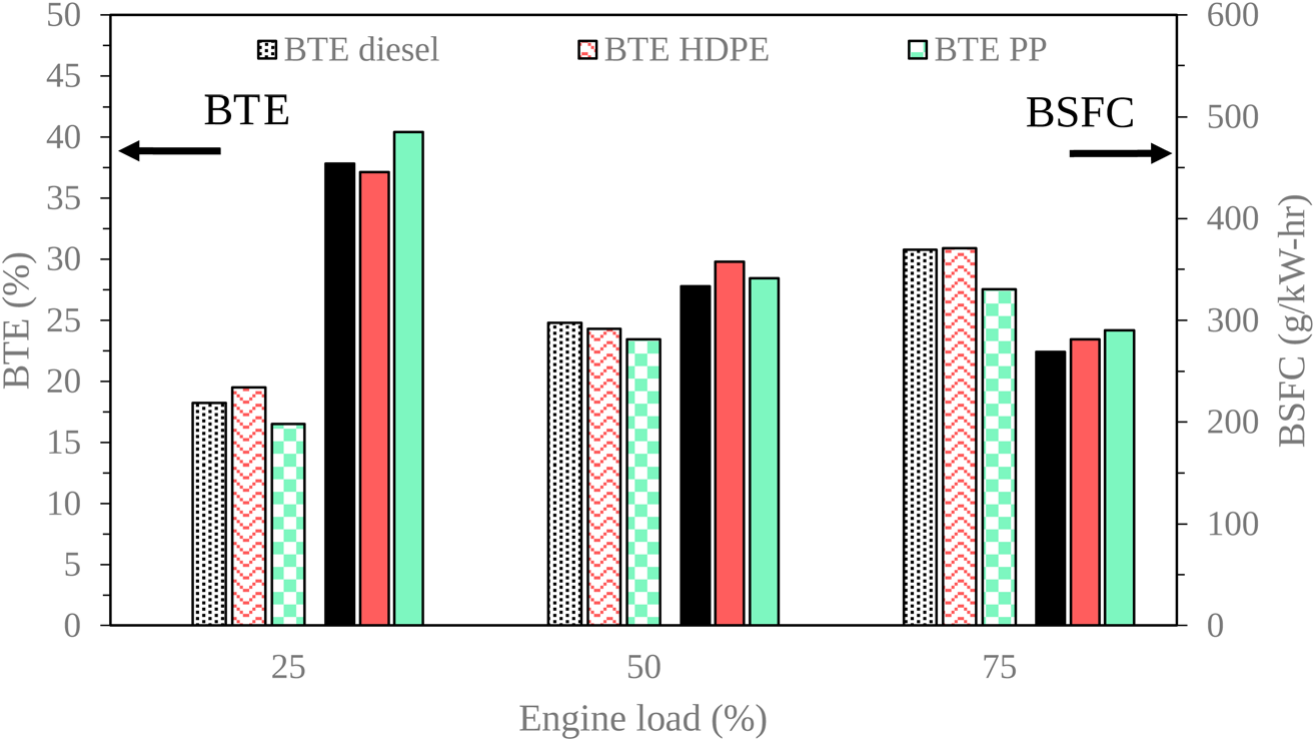
Variation of brake thermal efficiency and brake specific fuel consumption with engine load.

**Fig. 16:**
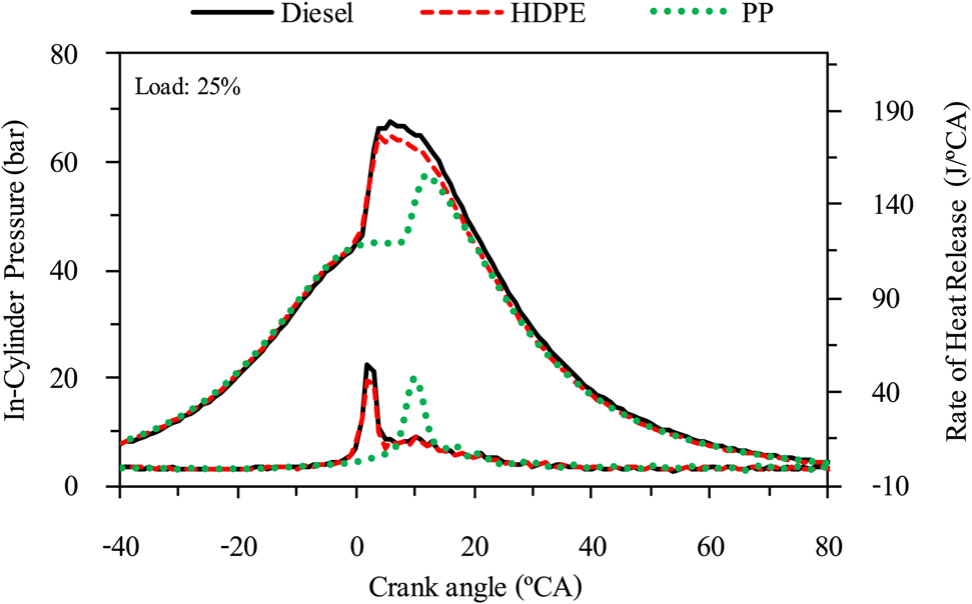
In-cylinder pressure and heat release rate at 25% engine load.

Engine performance is a vital indicator of an engine's operational capabilities. Brake Thermal Efficiency (BTE) serves as a key metric, denoting the engine's power output relative to the power consumed. Fig. 15 and 16 show variations of engine load with brake thermal efficiency and brake specific fuel consumption (BSFC), respectively. In this experimental evaluation, we noted a consistent upward trajectory in BTE across all test fuels as engine load increased. This phenomenon can be primarily attributed to the heightened combustion temperatures, resulting in improved energy conversion efficiency. It is noteworthy that PP exhibited a lower BTE compared to conventional diesel fuel across all engine load conditions. This variance can be attributed to the higher carbon compound composition of PP relative to diesel fuel, which increases the mass of the fuel–air mixture. Consequently, this elevation in mass leads to a higher heat release rate during combustion. However, it's important to acknowledge that PP also presents a longer ignition delay compared to diesel fuel. The high heat release rate with ignition delay can cause more heat loss during combustion.^30^ BTE exhibited by the engine when fueled with High-Density Polyethylene (HDPE) closely paralleled that of diesel fuel across all test loads. This similarity in BTE can be attributed to the comparable fuel properties of HDPE and diesel, resulting in similar combustion characteristics. Consequently, the BTE values for HDPE were consistently higher than those observed for PP across all load conditions.

Brake specific fuel consumption (BSFC) tended to decrease when increasing engine loads due to the increase in in-cylinder temperature enhanced fuel conversion to heat energy. PP provided higher BSCF than diesel and HDPE for all loads, due to heat losses in the combustion process.^30^ High-Density Polyethylene (HDPE) yielded marginally higher Brake Specific Fuel Consumption (BSFC) compared to diesel fuel under middle and high engine load conditions. This outcome can be attributed to HDPE's lower kinematic viscosity and lower cetane index. A lower cetane index adversely affects air–fuel mixing, while lower density increases fuel compressibility, resulting in an elevated injection volume. These factors collectively contribute to ignition occurring at an inappropriate position. In contrast, when the engine was fueled with PP, it exhibited higher BSFC than when fueled with HDPE. This can be attributed to increased heat losses during the combustion process and higher viscosity inherent to PP. Higher viscosity caused worse fuel atomization during injection, which resulted in poor energy conversion from fuel.^31^

### 3.4 Combustion characteristics

**Fig. 17:**
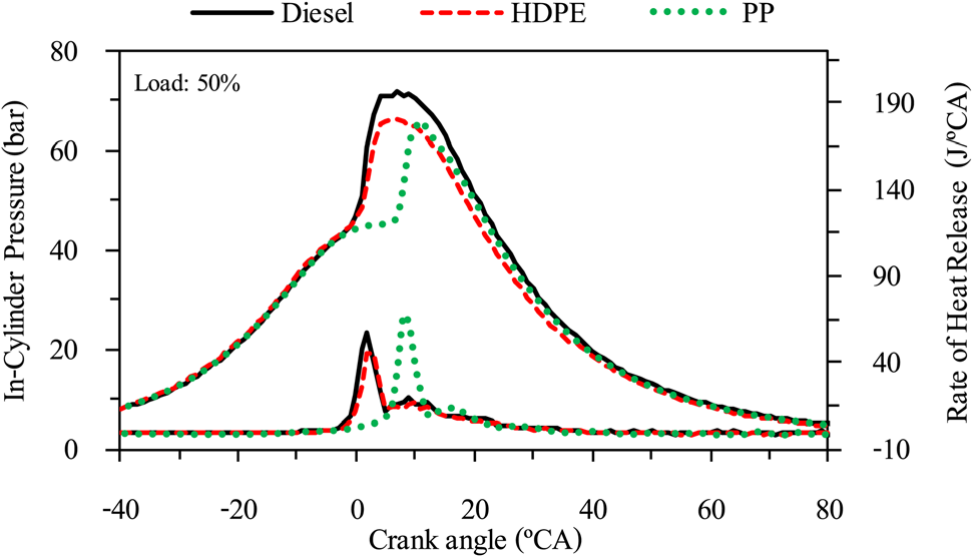
In-cylinder pressure and heat release rate at 50% engine load.

**Fig. 18:**
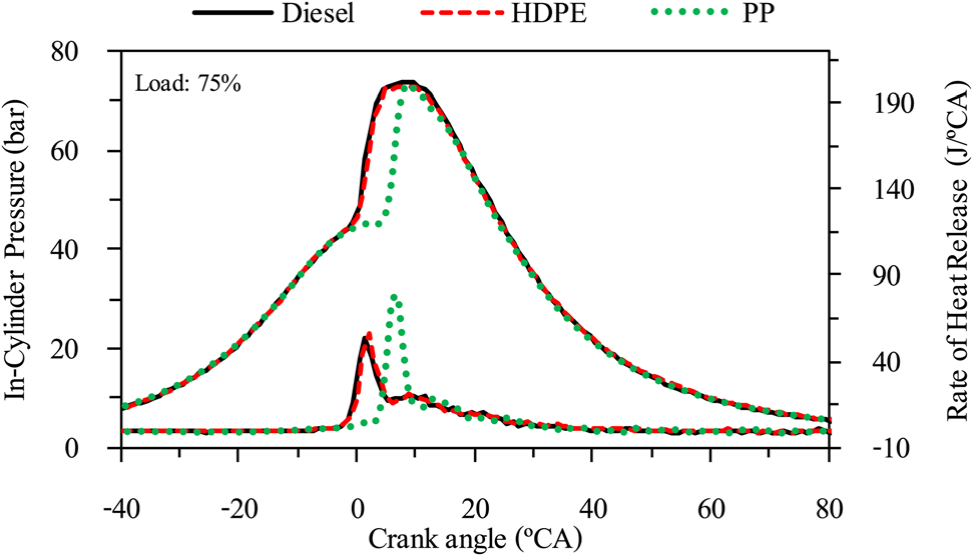
In-cylinder pressure and heat release rate at 75% engine load.

The combustion characteristics during the combustion process of the internal combustion engine are directly affected by fuel properties. The combustion characteristics of the test fuels in this study were investigated based on the cylinder pressure to analyze the combustion process. The cylinder pressure of 100 cycles was measured and averaged. In-cylinder pressure (ICP) and Heat release rate (HRR) are the two parameters that can be used to explain the combustion phenomenon. From Fig. 16–18 it can be observed that ICP and HRR of all test fuels increased as the engine load increased due to the higher engine loads demanding more power output, which necessitates an increase in fuel injection. When more fuel is injected into the combustion chamber during each cycle, it results in a larger and more energetic combustion event. This increased fuel quantity contributes significantly to the rise in in-cylinder pressure and the rate of heat release. Moreover, when engine load increases, the combustion process typically lasts longer, allowing for more complete burning of the air–fuel mixture. A longer combustion duration leads to a higher total heat release and increased in-cylinder pressure. HDPE provides lower ICP and HRR than diesel fuel for all engine loads due to its slightly lower cetane index and higher sulphur content.^32^ When the engine was fueled with low cetane index fuel, it caused worse air–fuel mixing, which provided a worse combustion process, and the higher sulphur content may disrupt combustion efficiency through the creation of sulphur compounds that have the potential to contaminate critical engine components such as fuel injectors and piston rings. Consequently, this interference can result in incomplete combustion, a decrease in engine power output, and alterations in both the pressure and heat release characteristics within the engine cylinder. From Fig. 16–18 it can be observed that PP fuel provides more retarded ignition than HDPE and a higher peak of HRR, which indicates that large quantities of fuel were ignited at the beginning of combustion. The reasons may be due to (1) lower oxygen content which cannot improve air fuel mixing, (2) higher kinematic viscosity which provides worse fuel atomization that needs more time to evaporate ^31^ and (3) lower density, which increases compressibility so that it needs more time to increase pressure in the fuel injection system, resulting in more delay to injecting the fuel, especially when a pump-line nozzle injection system is used with the test engine.

### 3.5 Emission characteristics

**Fig. 19:**
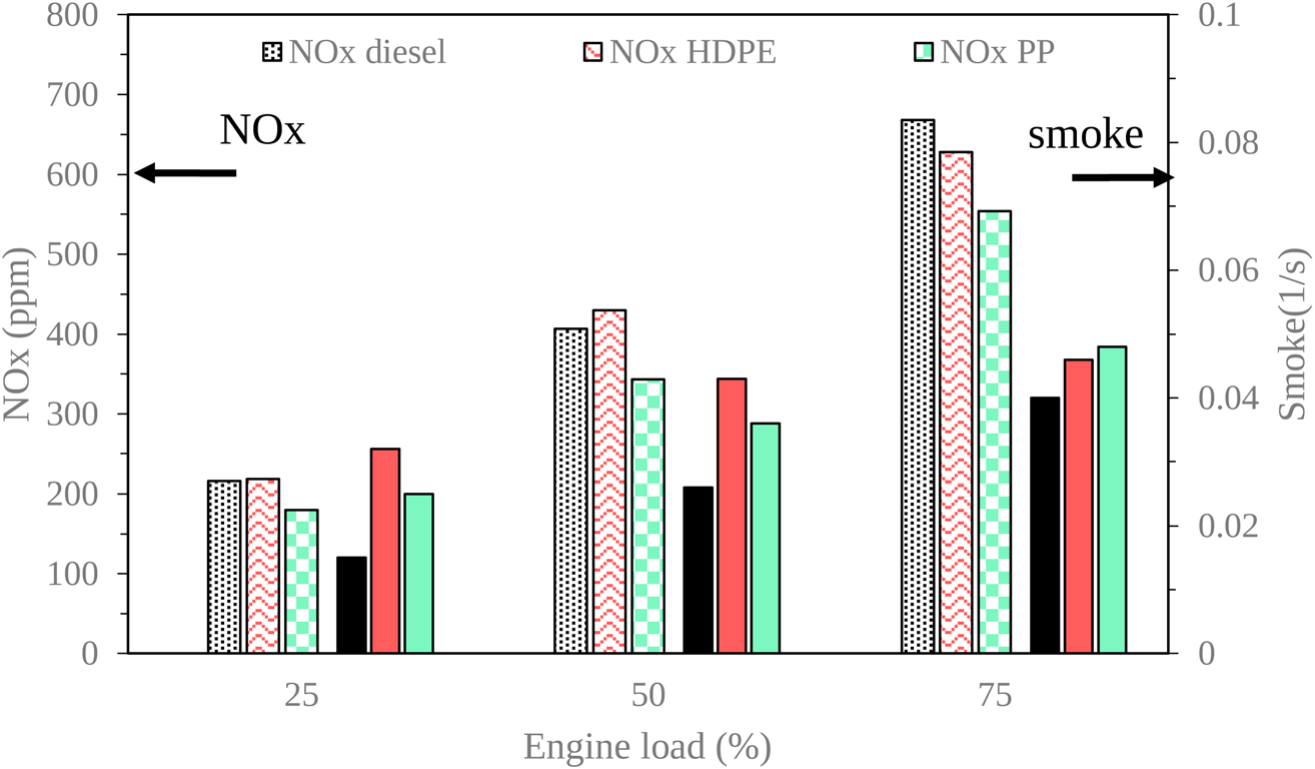
Variation of NO_*X*_ and smoke emission with engine load.

**Fig. 20:**
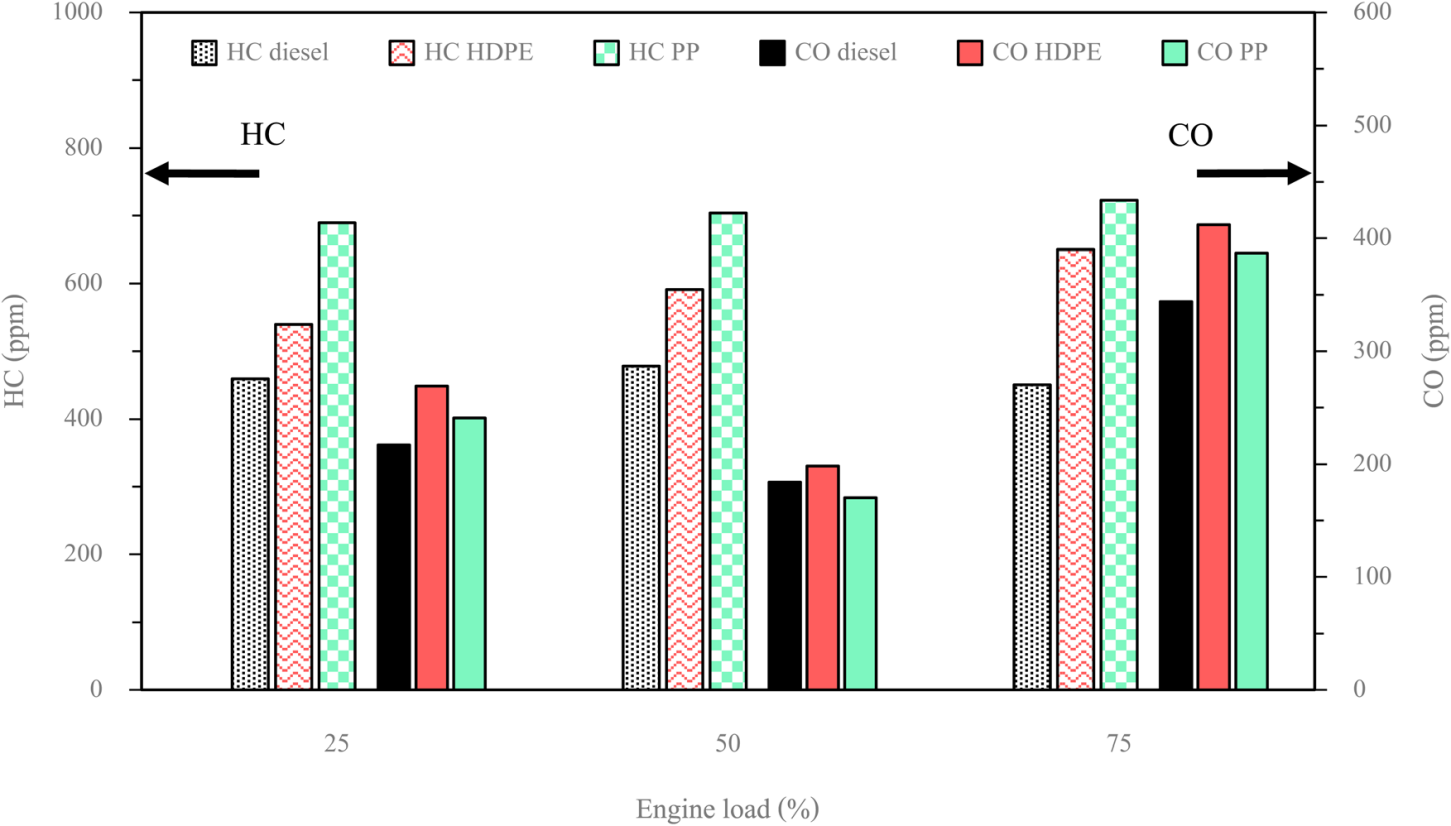
Variation of HC and CO emission with engine load.

The exhaust gas emissions, which consist of oxides of nitrogen (NO_*X*_), smoke, unburned hydrocarbon (HC) and carbon monoxide (CO) at all engine loads tested are shown in Fig. 19 and 20.

NO_*X*_ constitutes a primary pollutant emanating from diesel engines, primarily attributed to the operational characteristic of diesel engines, which involves the use of an excess amount of air.^8^ As depicted in Fig. 19, the NO_*X*_ emissions from all test fuels exhibited an increase with the rise in engine load. This phenomenon can be attributed to the elevated combustion temperature, which is conducive to the formation of thermal NO_*X*_.^33^ Under low and middle loads, HDPE exhibited higher NO_*X*_ emissions compared to diesel fuel that may be due to HDPE, typically exhibiting a higher hydrogen–carbon ratio compared to diesel fuel. The increased hydrogen content in the fuel can lead to more available hydrogen atoms for the formation of thermal NO_*X*_ during the combustion process, contributing to the observed higher NO_*X*_ emissions at both low and middle engine loads. Under high loads, HDPE exhibits lower NO_*X*_ emissions compared to diesel fuel, possibly attributable to its lower density, leading to higher compressibility and an increased fuel injection rate into the combustion chamber. The greater quantity of HDPE fuel injected causes a higher amount of soot and volatile organic material present in the combustion chamber that can absorb heat. In contrast, PP yields lower NO_*X*_ emissions than diesel and HDPE due to increased heat losses during the combustion process.

Smoke emissions from a diesel engine cause the visible presence of particulate matter or soot in the exhaust gases discharged by the engine during the combustion process. As illustrated in Fig. 19,^34^ it becomes evident that smoke emissions exhibit an upward trend with increasing engine operating loads, primarily when a rich air–fuel mixture is combusted, thus leading to the development of smoke precursor. In this context, both plastic oils, namely HDPE and PP, demonstrate higher levels of smoke emissions compared to conventional diesel fuel across all engine load conditions, owing to their lower density, which results in a greater volume of fuel being introduced into the combustion process. In a detailed comparison between HDPE and PP, it is observed that HDPE yields more pronounced smoke emissions than PP at lower and moderate engine loads. This observation can be attributed to HDPE having lower density, which contributes to an increased fuel supply to the engine, and higher sulphur content, which can promote the formation of sulfate particulate matter. These sulfate particulates contribute to the overall smoke emissions from the engine. Conversely, despite PP having higher ignition delay, its higher cetane index and viscosity promote improved air–fuel mixing, resulting in more complete combustion.

HC represents unburned fuel after the combustion process from the combustion chamber. Fig. 20 illustrates the trend in HC emissions for both plastic oils and diesel fuel as engine load varies. It is observed that HC emissions from plastic oils tend to increase with rising engine load, while diesel fuel has no clear trend. This increase in HC emissions can be attributed to insufficient time available for breaking down the hydrocarbon chains within the fuel molecules. Notably, both plastic oils consistently yield higher HC emissions than diesel fuel across all engine load conditions, due to their higher fuel supply for combustion, which results from their higher compressibility.^4^ In addition, the lower oxygen content of both plastic pyrolysis fuels compared with diesel fuel can result in unevenly distributed combustion. Furthermore, a comparison between HDPE and PP reveals that HC emissions from PP are greater than those from HDPE across all engine load conditions. This difference can be attributed to the higher carbon compound content and lower oxygen content in PP compared to HDPE, resulting in an increased mass of the fuel–air ratio. Consequently, this necessitates more time for the breakdown of chemical bonds within the fuel, leading to higher HC emissions.

CO emissions serve as an indicator of incomplete combustion. Fig. 20 illustrates that, among all the fuels tested, the highest CO emissions were observed under high engine load conditions, while the lowest CO emissions occurred during middle engine load conditions. During low engine load operation, the combustion temperature remains insufficient to break down fuel chemical bonds and convert them into CO_2_. Conversely, at middle engine load, a greater quantity of fuel is introduced into the combustion process, elevating the temperature to a level conducive for complete combustion, given the appropriate fuel–air mixture. Under high engine load conditions, an excessive fuel supply limits the time available for CO_2_ formation, despite the elevated combustion chamber temperature. It is worth noting that, in this context, the quantity of fuel supplied exerts a more significant influence on CO_2_ formation than temperature alone. The CO emission of HDPE was higher than diesel fuel for all engine loads due to lower in-cylinder pressure causing a lower combustion temperature that is unsuitable for CO_2_ formation. At a middle engine load, PP exhibits slightly lower CO emissions compared to diesel fuel. However, at both low and high engine loads, PP demonstrates higher CO emissions than diesel fuel. This phenomenon can be attributed to delayed ignition in PP, leading to inadequate time for complete combustion. Specifically, PP exhibits significant ignition retardation in comparison to diesel fuel, contributing to the observed increase in CO emissions. In contrast to PP, CO emissions from HDPE are elevated across all engine loads. This is attributed to a lower cetane index in HDPE, leading to suboptimal fuel–air mixing. Additionally, higher concentrations of C19–23 and C > 23 compounds in HDPE necessitate increased heat energy to break down chemical bonds and form CO_2_.

## 4 Conclusion

In summary, the plastic pyrolysis oils derived from HDPE and PP exhibit carbon range distributions within the diesel fuel range, possessing similar fuel properties. They can serve as alternative fuels in diesel engines without requiring any engine modification. On the contrary, PS, with a carbon distribution akin to gasoline, is unsuitable due to its fuel properties being aligned with gasoline. PP demonstrates superior fuel lubricity, attributed to higher kinematic viscosity and sulphur content, forming a protective film boundary. The lubricating ability of diesel fuel, particularly in palm biodiesel blends, is enhanced due to the ester functional group. HDPE, while providing similar brake thermal efficiency (BTE) to diesel, exhibits lower fuel lubricity due to a 10% lesser film percentage compared to PP. To enhance experiment accuracy, utilizing a closed HFRR specimen holder is recommended for PS lubricity tests. HDPE, offering comparable BTE to diesel, displays similar combustion characteristics, albeit with slightly lower in-cylinder pressure and heat release rate. In contrast, PP exhibits significant combustion retardation owing to having lower oxygen content, higher viscosity and lower density. HDPE yields higher NO_*X*_ emissions than diesel at low and medium engine loads, but lower emissions at high loads. PP, with lower NO_*X*_ emissions, surpasses both HDPE and diesel due to heat losses during combustion. Higher hydrocarbon (HC) emissions from both plastic oils compared to diesel are noted, with HDPE's HC increase attributed to lower oxygen content at low loads and increased fuel supply at higher loads. CO emissions are higher in HDPE than diesel across all loads, while PP exceeds diesel at low and high loads. HDPE's CO levels surpass PP due to a lower cetane index and higher heavy composition. Smoke emissions from HDPE and PP are elevated, influenced by increased fuel supply, higher sulphur content, and lower oxygen content, leading to sulphurate particulate matter with low soot oxidation. Forthcoming investigations into plastic pyrolysis oil will prioritize enhancing its purity to optimize its compatibility with engine systems. This initiative aims to refine the oil's properties for improved engine performance. Additionally, a comprehensive long-term engine testing phase will be undertaken to assess the sustained effects on engine functionality following the utilization of plastic pyrolysis oil.

## Conflicts of interest

There are no conflicts to declare.

## Supplementary Material

## References

[cit1] Kosior E. and Mitchell J., Current Industry Position on Plastic Production and Recycling, Plastic Waste and Recycling, 2020, pp. 133–162

[cit2] Kaimal V. K., Vijayabalan P. (2017). A detailed investigation of the combustion characteristics of a DI diesel engine fuelled with plastic oil and rice bran methyl ester. J. Energy Inst..

[cit3] Gug J., Cacciola D., Sobkowicz M. J. (2015). Processing and properties of a solid energy fuel from municipal solid waste (MSW) and recycled plastics. Waste Manage..

[cit4] Kaimal V. K., Vijayabalan P. (2016). A study on synthesis of energy fuel from waste plastic and assessment of its potential as an alternative fuel for diesel engines. Waste Manage..

[cit5] Ahmad I., Khan M. I., Khan H., Ishaq M., Tariq R., Gul K. (2014). *et al.*, Pyrolysis Study of Polypropylene and Polyethylene Into Premium Oil Products. Int. J. Green Energy.

[cit6] Damodharan D., Sathiyagnanam A. P., Rana D., Rajesh Kumar B., Saravanan S. (2017). Extraction and characterization of waste plastic oil (WPO) with the effect of n -butanol addition on the performance and emissions of a DI diesel engine fueled with WPO/diesel blends. Energy Convers. Manage..

[cit7] Mariappan M., Panithasan M. S., Venkadesan G. (2021). Pyrolysis plastic oil production and optimisation followed by maximum possible replacement of diesel with bio-oil/methanol blends in a CRDI engine. J. Cleaner Prod..

[cit8] Arjharn W., Liplap P., Maithomklang S., Thammakul K., Chuepeng S., Sukjit E. (2022). Distilled Waste Plastic Oil as Fuel for a Diesel Engine: Fuel Production, Combustion Characteristics, and Exhaust Gas Emissions. ACS Omega.

[cit9] Gollakota A. R. K., Reddy M., Subramanyam M. D., Kishore N. (2016). A review on the upgradation techniques of pyrolysis oil. Renewable Sustainable Energy Rev..

[cit10] Anuar Sharuddin S. D., Abnisa F., Wan Daud W. M. A., Aroua M. K. (2016). A review on pyrolysis of plastic wastes. Energy Convers. Manage..

[cit11] Abnisa F., Wan Daud W. M. A. (2014). A review on co-pyrolysis of biomass: An optional technique to obtain a high-grade pyrolysis oil. Energy Convers. Manage..

[cit12] Mastral F., Esperanza E., Garcıa P., Juste M. (2002). Pyrolysis of high-density polyethylene in a fluidised bed reactor. Influence of the temperature and residence time. J. Anal. Appl. Pyrolysis.

[cit13] Marcilla A., Beltrán M. I., Navarro R. (2009). Thermal and catalytic pyrolysis of polyethylene over HZSM5 and HUSY zeolites in a batch reactor under dynamic conditions. Appl. Catal. B: Environ..

[cit14] Bagri R., Williams P. T., pyrolysis a (2002). Catalytic pyrolysis of polyethylene. J. Anal. Appl. Pyrolysis.

[cit15] Demirbas A. (2004). Pyrolysis of municipal plastic wastes for recovery of gasoline-range hydrocarbons. J. Anal. Appl. Pyrolysis.

[cit16] FakhrHoseini S. M., Dastanian M. (2013). Predicting Pyrolysis Products of PE, PP, and PET Using NRTL Activity Coefficient Model. J. Chem..

[cit17] Onwudili J. A., Insura N., Williams P. T. (2009). Composition of products from the pyrolysis of polyethylene and polystyrene in a closed batch reactor: Effects of temperature and residence time. J. Anal. Appl. Pyrolysis.

[cit18] Maithomklang S., Sukjit E., Srisertpol J. (2020). Experimental investigation of ethanol blended with waste plastic oil as an alternative biofuel in a diesel engine. Suranaree J. Sci. Technol.

[cit19] Kaimal V. K., Vijayabalan P. (2016). An investigation on the effects of using DEE additive in a DI diesel engine fuelled with waste plastic oil. Fuel.

[cit20] Karisathan S. N., Ramachandran B. A. (2016). Experimental Investigation on Thermocatalytic Pyrolysis of HDPE Plastic Waste and the Effects of Its Liquid Yield over the Performance, Emission, and Combustion Characteristics of CI Engine. Energy Fuels.

[cit21] Kalargaris I., Tian G., Gu S. (2017). Experimental evaluation of a diesel engine fuelled by pyrolysis oils produced from low-density polyethylene and ethylene–vinyl acetate plastics. Fuel Process. Technol..

[cit22] Singh T. S., Verma T. N., Singh H. N. (2020). A lab scale waste to energy conversion study for pyrolysis of plastic with and without catalyst: Engine emissions testing study. Fuel.

[cit23] Singh T. S., Rajak U., Dasore A., Muthukumar M., Verma T. N. (2021). Performance and ecological parameters of a diesel engine fueled with diesel and plastic pyrolyzed oil (PPO) at variable working parameters. Environ. Technol. Innovation.

[cit24] Kalargaris I., Tian G., Gu S. (2018). Experimental characterisation of a diesel engine running on polypropylene oils produced at different pyrolysis temperatures. Fuel.

[cit25] Miranda R., Pakdel H., Roy C., Vasile C. (2001). Vacuum pyrolysis of commingled plastics containing PVC II. Product analysis. Polym. Degrad. Stab..

[cit26] Jahirul M. I., Faisal F., Rasul M. G., Schaller D., Khan M. M. K., Dexter R. B. (2022). Automobile fuels (diesel and petrol) from plastic pyrolysis oil—Production and characterisation. Energy Rep..

[cit27] EN ISO 12156-1, Diesel Fuel: Assessment of Lubricity Using the High Frequency Reciprocating Rig (HFRR)-Part 1: Test Method, European Committee for Standardization, Brussels, Belgium, 2018

[cit28] Jenkins R. W., Moore C. M., Semelsberger T. A., Chuck C. J., Gordon J. C., Sutton A. D. (2016). The Effect of Functional Groups in Bio-Derived Fuel Candidates. ChemSusChem.

[cit29] Sukjit E., Poapongsakorn P., Dearn K. D., Lapuerta M., Sánchez-Valdepeñas J. (2017). Investigation of the lubrication properties and tribological mechanisms of oxygenated compounds. Wear.

[cit30] Mangesh V. L., Padmanabhan S., Tamizhdurai P., Ramesh A. (2020). Experimental investigation to identify the type of waste plastic pyrolysis oil suitable for conversion to diesel engine fuel. J. Cleaner Prod..

[cit31] Kaewbuddee C., Sukjit E., Srisertpol J., Maithomklang S., Wathakit K., Klinkaew N. (2020). *et al.*, Evaluation of Waste Plastic Oil-Biodiesel Blends as Alternative Fuels for Diesel Engines. Energies.

[cit32] Alam M., Song J., Acharya R., Boehman A., St. Miller K. J. (2004). Combustion and emissions performance of low sulfur, ultra low sulfur and biodiesel blends in a DI diesel engine. SAE Technical Paper.

[cit33] Varatharajan K., Cheralathan M. (2013). Effect of aromatic amine antioxidants on NOx emissions from a soybean biodiesel powered DI diesel engine. Fuel Process. Technol..

[cit34] Wathakit K., Sukjit E., Kaewbuddee C., Maithomklang S., Klinkaew N., Liplap P. (2021). *et al.*, Characterization and Impact of Waste Plastic Oil in a Variable Compression Ratio Diesel Engine. Energies.

